# COVID-19-Induced Diabetes Mellitus: Comprehensive Cellular and Molecular Mechanistic Insights

**DOI:** 10.3390/pathophysiology31020016

**Published:** 2024-04-08

**Authors:** Praise Tatenda Nhau, Mlindeli Gamede, Ntethelelo Sibiya

**Affiliations:** 1 Pharmacology Division, Faculty of Pharmacy, Rhodes University, Makhanda 6139, South Africa; tatenhau@gmail.com; 2 Human Physiology Department, University of Pretoria, Pretoria 0028, South Africa; mlindeli.gamede@up.ac.za

**Keywords:** diabetes, COVID-19, SARS-CoV-2, insulin resistance, glucose handling, metabolic pathways, inflammation

## Abstract

Despite evidence demonstrating the risks of developing diabetes mellitus because of SARS-CoV-2, there is, however, insufficient scientific data available to elucidate the relationship between diabetes mellitus and COVID-19. Research indicates that SARS-CoV-2 infection is associated with persistent damage to organ systems due to the systemic inflammatory response. Since COVID-19 is known to induce these conditions, further investigation is necessary to fully understand its long-term effects on human health. Consequently, it is essential to consider the effect of the COVID-19 pandemic when predicting the prevalence of diabetes mellitus in the future, especially since the incidence of diabetes mellitus was already on the rise before the pandemic. Additional research is required to fully comprehend the impact of SARS-CoV-2 infection on glucose tolerance and insulin sensitivity. Therefore, this article delves deeper into the current literature and links the perceived relationship between SARS-CoV-2 and diabetes. In addition, the article highlights the necessity for further research to fully grasp the mechanisms that SARS-CoV-2 utilises to induce new-onset diabetes. Where understanding and consensus are reached, therapeutic interventions to prevent the onset of diabetes could be proposed. Lastly, we propose advocating for the regular screening of diabetes and pre-diabetes, particularly for the high-risk population with a history of COVID-19 infection.

## 1. Introduction

The COVID-19 pandemic created a significant public health crisis, with long-term socioeconomic consequences, including the exacerbated burden on public health systems due to increased incidences of other non-communicable diseases such as diabetes mellitus (DM) [1]. Studies indicate that DM is one of the primary causes of both morbidity and mortality in developing countries such as South Africa [1]. Globally, it is projected that the prevalence of diabetes will increase from 415 million individuals in 2015 to 642 million individuals by the year 2040, indicating a significant rise in the burden of diabetes [2]. With the prevalence of DM increasing worldwide, it is prudent to explore the pathophysiology involved in COVID-19-induced DM. Risk factors of DM, such as sedentary lifestyles, high-fat diets, unbalanced diets, and obesity, contributing to insulin resistance and beta cell (β-cell) failure, have heightened during this COVID-19 era. However, the elusive phenomenon is how much damage the COVID-19 pandemic has left behind.

Diabetes mellitus refers to a group of chronic endocrine metabolic disorders that are characterised by a loss of glycaemic control, leading to elevated blood glucose concentration due to either insulin resistance or inadequate insulin production [3]. The hallmark symptoms of DM include polydipsia, polyphagia, and polyuria [4]. DM is classified into different categories. The main two types are type 1 diabetes mellitus (T1DM), which is characterised by insulin dependence, and type 2 diabetes mellitus (T2DM), which is characterised by insulin resistance [5]. T1DM is a chronic autoimmune disorder that results in the destruction of pancreatic beta cells in the islets of Langerhans. This results in a deficiency of insulin production and, subsequently, hyperglycaemia [3]. The primary aetiology has been attributed to the infiltration of the pancreas by T-cells [6]. In an inflammatory environment, insulitis is created as the disease progresses due to immune cells infiltrating the pancreas and targeting the insulin-producing cells [6]. T2DM, on the other hand, is a chronic metabolic disorder characterised by poor regulation of carbohydrate and lipid metabolism, beta cell dysfunction, and insulin resistance, leading to glucose intolerance [3]. Glycaemic control is mostly maintained by insulin at the beginning of the disease [7]. When glucose tolerance diminishes, defects in β-cell function occur; thus, they function abnormally by the time diabetes becomes apparent [7]. Due to this desensitisation of the β-cells to insulin, it unfortunately compromises the target tissues. Fazakerley et al. reported that these target tissues, such as muscles and adipose, gradually become insensitive to insulin [8].

Literature evidence has linked DM with susceptibility to SARS-CoV-2 and developing severe COVID-19 complications [9,10]. On the other hand, COVID-19 has been associated with diabetes induction. In 2003, amidst the outbreak of SARS-CoV-1, which was described by Pal and Banerjee et al. as “the cousin” of COVID-19, a study was conducted on 39 patients who had no previous history of diabetes [9]. The results of this study showed that during their hospitalisation, 20 of these patients developed DM. Despite glycaemic management during the three years of follow-up, two of those patients had persistent DM [9]. Since this occurred due to the less virulent and less efficiently transmitted SARS-CoV-1, it is therefore not far-fetched to contend that the impact of SARS-CoV-2 would be far greater.

**Figure 1:**
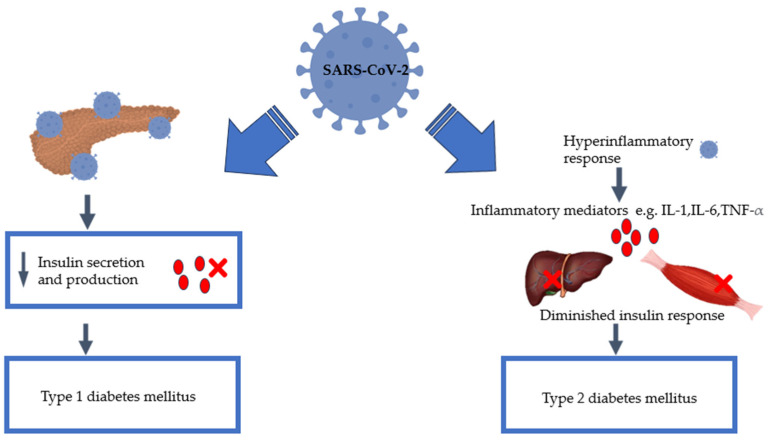
COVID-19 intersects with both type 1 diabetes mellitus and type 2 diabetes mellitus, illustrating the available potential mechanisms that may precipitate an increased risk of the development of new-onset DM for individuals who contracted the SARS-CoV-2 virus [2,6,7,8,9,10].

Indeed, COVID-19 has been associated with glycaemic aberrations, with reports of hospitalised patients demonstrating hyperglycaemia [11,12]. Despite this knowledge, there is still a paucity of consensus on the mechanisms associated with SARS-CoV-2-induced glycaemic abnormalities. Despite the differences in disease progression between T1DM and T2DM, there has been a convergence with respect to COVID-19 infection, as shown in Figure 1 below. In this review paper, our efforts are directed at shedding possible key cellular and molecular links between COVID-19 and diabetes by consolidating evidence from the current research in this area.

## 2. SARS-CoV-2 Infection

Severe acute respiratory syndrome coronavirus 2 (SARS-CoV-2) is a highly infectious novel coronavirus that emerged in 2019, causing a global pandemic of respiratory illness known as coronavirus disease 2019 (COVID-19) [13]. Coronaviruses belong to a vast group of RNA viruses enclosed in a membrane envelope [14,15]. This virus is also a single-stranded positive-sense RNA virus that infects both humans and animals such as bats [14,16]. The transmission of this virus occurs when an infected person releases respiratory droplets that contain the virus, and they enter the orifices, such as the nose and mouth, to ultimately reach the respiratory tract. SARS-CoV-2 enters the host cell by binding to angiotensin-converting enzyme 2 (ACE 2) via the spike protein. ACE2 is the functional receptor for the spike protein in SARS-CoV-2 that is found primarily in the upper respiratory tract, heart, endothelial cells, pancreas, kidney tubular epithelium, and enterocytes [16,17,18]. This explains why the effects of SARS-CoV-2 are widespread in the body. Research has shown that SARS-CoV-2 can infiltrate the cells that possess ACE2 receptors, and it cannot enter cells that express different coronavirus receptors like aminopeptidase N and dipeptidyl peptidase 4 (DPP4) [19]. Surface S (spike) proteins have a receptor-binding domain that initiates the endocytosis of virions. Once the virus enters the host cell, it hijacks the host cell machinery to produce more virions. The translation of viral proteins and copying of the genome are both required to create new virions.

Since December 2019, millions of individuals worldwide have been impacted by the COVID-19 pandemic [13]. SARS-CoV-2 had such a profound global impact that millions of lives were lost. As of 21 June 2023, the World Health Organisation (WHO) reported 768,187,096 confirmed cases of COVID-19 globally and a staggering 6,945,714 deaths [20]. Although these numbers are significantly high, they unfortunately do not account for unreported cases and deaths. The data available only informs us about recorded infections and deaths but provides no information on the long-term effects of COVID-19, such as “long COVID-19”. Owing to this, we can safely say that the totality of the damage caused by the COVID-19 pandemic is still unknown. Coronaviruses have been responsible for three epidemics in the last 15 years. Approximately 26 countries were affected by the SARS-CoV-1 epidemic that occurred in 2003 in Guangdong, China [15]. The largest outbreak was the most recent one, which started in 2019.

## 3. SARS-CoV-2 Infection and Diabetes Mellitus

Various studies have reported enhanced fatality in diabetic subjects who were infected with COVID-19 [13,21,22,23]. Those with comorbidities, such as diabetes mellitus, have been demonstrated in studies to be more vulnerable to catastrophic COVID-19 results [13,22,23]. Studies have also observed that elevated levels of glucose directly promote the replication of the SARS-CoV-2 virus in human monocytes [24]. Diabetes mellitus was linked to the first three deaths caused by COVID-19 in Hong Kong’s initial wave [25]. Wu et al. conducted a study on 52 individuals in intensive care and discovered that among the 32 individuals who did not survive, 22% had diabetes as a comorbidity [22]. In a separate study conducted by Yang et al., the authors found that the incidence of diabetes among the patients in intensive care was twice as high compared to both the non-intensive care patients and those with COVID-19 [23]. Similarly, in a study by Yang and colleagues (2020), 62% of ICU patients had diabetes, showing that diabetic patients predominate among ICU admissions [21]. For heightened compression, in the next section, we will provide an overview of the key glucose-handling process to contextualise SARS-CoV-2-induced hyperglycaemia or diabetes. Subsequently, we will explore the cellular and molecular mechanisms linking SARS-CoV-2 infection with the risk of the development of DM.

Insulin was used to treat patients with T2DM during the COVID-19 era. Since then, there have been different retrospective studies conducted that have implicated insulin as the causative agent for the increase in mortality, while others have disagreed. A retrospective study carried out by Yu et al. presented findings that indicated a notable increase in mortality among individuals with both COVID-19 and T2DM who were undergoing insulin therapy. This association was observed regardless of the severity of the COVID-19 infection [26]. Riahi et al. also supported the aforementioned findings [27]. The authors reported a significant association between inpatient mortality and peak insulin requirements. It was found that, despite patients having comparable steroid doses and glycated haemoglobin levels, the hospitalised patients who died used significantly higher insulin doses [27]. Furthermore, a meta-analysis of six studies conducted by Wang et al. supports this hypothesis, as the results unveiled a connection between insulin usage and higher mortality rates in individuals with both diabetes and COVID-19 [28]. Yang et al. also conducted a meta-analysis of eighteen articles and concluded three main things. Firstly, insulin treatment in COVID-19 patients resulted in a significantly higher mortality rate and COVID-19 complications. Furthermore, there was an observed trend that indicated an increased likelihood of hospital admission for patients with both COVID-19 and diabetes [29].

However, there has been resistance to this hypothesis as other authors have stated that confounding variables may be attributed to this noted trend. The findings by Sardu et al. contradicted previous findings, as the researchers reported that insulin infusions may improve patient outcomes and help them achieve their glycaemic targets [30]. This comes after the patient group receiving insulin infusions experienced a significant reduction in plasma glucose after the treatment period compared to the patient group without the insulin infusion [30]. From our perspective, an increase in insulin in COVID-19 patients could allude to a state of insulin resistance, possibly instigated by the inflammatory state induced by SARS-CoV-2 infection. However, we also cannot rule out psychological stress, which could also favour an insulin resistance state, therefore necessitating higher insulin dose requirements. Table 1 provides a summary of studies supporting the directional relationship between COVID-19 and diabetes development.

## 4. Insulin Signalling Pathway: Effect of SARS-CoV-2

Insulin plays a pivotal role in promoting glucose uptake primarily by translocating GLUT4 from an intracellular storage reservoir to the cell’s plasma membrane. When insulin enters the bloodstream, insulin-receptor complexes are formed in a few target tissues, such as adipose and skeletal muscle, to elicit a biological response [36]. When insulin binds to its tyrosine kinase receptor on the cell surface, it triggers a conformational change that results in the autophosphorylation of the tyrosine residues in the intracellular domain of the receptor [37,38,39]. Phosphorylated tyrosine residues on the receptor create a molecular interaction site for insulin receptor substrate (IRS) proteins. The IRS proteins activate phosphatidylinositol-3-kinase (PI3K) [38]. This enzymatic interaction stimulates the production of phosphatidylinositol (3,4,5)-trisphosphate (PIP3). PIP3 subsequently activates protein kinase B (Akt). When Akt is activated, it promotes the translocation of GLUT4 to the plasma membrane, which in turn facilitates insulin-driven glucose uptake and glycogen synthesis in the skeletal muscle and liver [39].

The phospho-inositol kinase, protein kinase B (AKT), and GLUT-4 proteins play essential roles in controlling glucose metabolism and insulin signalling pathways. In cases such as obesity or insulinoma, there is a downregulation of insulin receptors, resulting in insulin resistance and, ultimately, type 2 diabetes mellitus [40].

Studies have shown that SARS-CoV-2 proteins manipulate host signalling pathways, creating an environment favourable for viral replication [41]. Approximately 100 human kinases involved in cellular physiology, metabolism, and immune responses can be upregulated or downregulated by SARS-CoV-2 proteins [41]. Shin et al. played a pivotal role in presenting one of the initial scientific findings that demonstrated how SARS-CoV-2 disrupts the insulin/insulin-like growth factor (IGF) signalling pathway in the respiratory tract, and other metabolic and endocrine tissues such as the liver, adipose tissue, and pancreatic tissues [42]. As previously mentioned, the insulin signalling pathway is critical for glucose homeostasis. Disruptions in the insulin signalling pathway can lead to both T1DM and T2DM. In T1DM, β-cell destruction leads to the disruption of the insulin signalling pathway, whereas with T2DM, the key insulin-sensitive cells become resistant to the effects of insulin, therefore impairing the signalling pathway’s ability to effectively maintain glucose homeostasis [5,6]. While the specific mechanisms differ between the two types, COVID-19-induced disruptions in the insulin signalling pathway can be central to the development and propagation of both T1DM and T2DM. The authors reported that SARS-CoV-2 infection resulted in the downregulation of genes significantly associated with insulin signalling, mTOR signalling, and MAPK signalling [42]. The authors also revealed that there was a diverse array of deficiencies in the insulin/IGF signalling components, encompassing disruptions in the insulin receptor substrate (IRS), PI3K, AKT, and mTOR molecules. As also described in the insulin signalling pathway, these proteins are essential for the maintenance of cellular homeostasis.

The mechanism of the impairment of the insulin/IGF signalling pathway was seen to be facilitated by heightened interferon responses, especially the upregulation of interferon regulatory factor-1 (IRF-1) [42,43]. This was also an observation that Sestan et al. shared in their proposed model of how viruses potentially trigger new-onset diabetes [44]. IRF-1 can both activate and repress gene transcription to finely tune the immune response, ensuring an appropriate and controlled reaction to various stimuli. A SARS-CoV-2 infection was reported to cause an increase in the gene expression of IRF-1, which mediated impairments in the insulin/IGF signalling pathway [42]. The insulin/IGF signalling pathway is important in various biological processes, including energy metabolism and the preservation of cell viability. This dysfunction of the insulin/IGF signalling pathway in metabolic organs and tissues can lead to insulin resistance, cellular death, and metabolic abnormalities. This, in turn, contributes to the development of various metabolic disorders, including hyperglycaemia, diabetes, hyperlipidaemia, and obesity [42]. Additional clinical research is imperative to address these aspects to prevent new-onset diabetes and other complications that may arise from future coronaviruses.

## 5. Virus-Induced Glucose Homeostasis Alteration

Viruses activate host signalling pathways from the moment they attach to host cells. This marks the earliest interaction between the virus and the host [45]. Viruses manipulate host metabolism by disrupting essential metabolic pathways, such as the PI3K/AKT pathway, and targeting key regulatory proteins [46]. Metabolic signalling pathways serve as primary decision-making processes that coordinate cell signalling, gene transcription, and precise modulation, all of which are crucial for organism survival. Viral adaptation has enabled viruses to target these pathways, modifying cellular metabolism to their advantage [45]. To facilitate replication, the virus utilises the host’s metabolic activity, which explains why it induces a reprogramming of the cell’s metabolism [47]. Viruses have been shown to alter glucose metabolism in host cells through various mechanisms, resulting in the precipitation of T1DM and T2DM. Fontaine et al. reported that dengue virus (DENV) infection in primary human foreskin fibroblast (HFF) cells results in an amplification of glycolysis and changes in the levels of glycolytic intermediates when compared to uninfected cells [48]. Cells infected with human cytomegalovirus (HCMV) have been reported to present a heightened reliance on glucose, leading to an upregulation of glycolysis and increased production of lactate [47]. The infected cells also promote glycolysis by activating AMP-activated protein kinase. A substantial body of circumstantial evidence has implicated enteroviruses, specifically coxsackieviruses, as prime viral candidates responsible for precipitating T1DM [49]. Research conducted in 1978 by Yoon et al. showed that certain inbred strains of mice can be infected by Coxsackie virus B4, and the virus can destroy the pancreatic beta cells, thus precipitating hypoinsulinaemia and subsequently leading to T1DM [50]. Support for the conclusions drawn in the previous study was reinforced by Dotta et al., who provided compelling evidence that revealed the direct involvement of the Coxsackie B4 enterovirus and pancreatic β cells of individuals with type 1 diabetes [51]. This viral infection was shown to induce inflammation and functional impairment in T1DM patients. In addition to coxsackievirus, rotaviruses have also been implicated in T1DM development. Honeyman et al. reported that rotaviruses may exacerbate pancreatic islet autoimmunity and thus cause T1DM [52].

Viral infections have also been associated with the development of T2DM. Yang et al. conducted a study comparing SARS patients without a history of T2DM. In their investigation over a 3-year follow-up, more than 50% of the patients developed diabetes while in hospital for SARS-CoV-1 infection. However, after three years of recovery from the viral infection, only 5% of patients remained diabetic [23]. Considering the similarities between SARS-CoV-1 and SARS-CoV-2, it is highly likely that new-onset diabetes would also be a manifestation of long COVID-19.

In pre-diabetic individuals, Sestak et al. highlighted a potential mechanism by which viral inflammation can lead to T2DM in a pre-diabetic mouse model [44]. By exposing the pre-diabetic mice to different viral pathogens, the researchers reported that viral infections independently pose a risk for the onset of T2DM in pre-diabetic obese mice. Viral-induced interferon-γ has been recognised as the culprit in the emergence of insulin resistance in the skeletal muscle, especially in the context of obesity. Given the important role of skeletal muscles in maintaining normoglycemia and the widespread occurrence of insulin resistance in individuals with obesity, it becomes prudent to combat these effects in humans [44]. With this background, we will now consider unpacking plausible mechanistic insights associated with SARS-CoV-2-induced glycaemic abnormalities.

## 6. Pancreatic Damage: Effect of SARS-CoV-2

Although the respiratory system is the major target of SARS-CoV-2, there is accumulating evidence that the virus can also impact other organ systems, including the endocrine system [53]. COVID-19 may directly or indirectly damage other systemic organs, including the pancreas. This can be via direct invasion by SARS-CoV-2 via the ACE2 receptor or indirect damage caused by the cytokine storm [54]. Interestingly, clinical data established that normal individuals possess ACE2 in their pancreas, which was marginally higher compared to that in the lungs. Kelesidis et al. reported that SARS-CoV-2 can directly invade pancreatic endocrine cells and trigger indirect responses within the body, such as autoimmune and inflammatory reactions, which may contribute to additional harm to the endocrine cells [43]. This was further supported by reports by Liu et al., where patients with severe COVID-19 presented with pancreatic injury [54,55]. Valuable evidence suggests that SARS-CoV-2 infects and replicates in the cells of the human endocrine and exocrine pancreas [32,56,57]. A study conducted by Müller et al. revealed that SARS-CoV-2 induces changes in the morphology and function of human pancreatic cells such as the β-cells. This results in impaired glucose-stimulated insulin secretion and a reduction in the insulin-secretory granules [32]. Similarly, Morris et al. also reported that SARS-CoV-2 replicates within the islets of Langerhans, thus causing disruptions in insulin secretion [56]. Qadir et al. provided additional evidence that potentially implicates SARS-CoV-2 infection and new-onset diabetes [57]. The above literature evidence suggests that SARS-CoV-2 may trigger both acute and chronic pancreatic dysfunction directly through pancreatic invasion [57]. Furthermore, emerging evidence has been observed that COVID-19 is associated with autoimmunity, as anti-pancreatic antibodies have been observed [54]. The entirety of these findings suggests that SARS-CoV-2 can potentially contribute to the metabolic dysregulation observed in patients with COVID-19. Pancreatic damage, particularly in β-cells, is a plausible explanation linking COVID-19 with acute hyperglycaemia and T1DM.

T1DM has been studied in the past as far back as 1991, and indisputable evidence suggests that viruses are aetiologic agents. A study conducted by Wagenknecht et al. revealed that there was a statistically significant increase in the incidence of T1DM following an epidemic of Coxsackievirus B5 [58]. The study explored several possible mechanisms through which T1DM could be precipitated by the presence of the virus, and these were similar to what we observed with COVID-19. The mechanisms observed all focused on the destruction of β-cells. The simplest of the three was via direct action on the cells, whereas the other two relied on either an immune recognition of altered β-cells or the virus acting as a trigger for genetically susceptible individuals [58]. This study was conducted in Alabama, and the researchers found that their results were consistent with findings from other regions, such as the US Virgin Islands and Poland [58]. A recent study in 2018 confirmed previous observations that enterovirus infections (including CVB) may increase diabetes type 1 islet autoimmunity [59]. A significant proportion of type 1 diabetics had the enterovirus in their pancreatic β-cells, suggesting a persistent viral infection in these individuals [59]. This might be the case with COVID-19, and thus, more research is warranted to elucidate the clinical significance of the involvement of the pancreas in COVID-19 patients.

## 7. SARS-CoV-2-Induced Glycolytic and Hepatic Gluconeogenic Pathways

Post-mortem studies in patients infected with SARS-CoV-2 have revealed clues indicating traces of viral proteins, which could be indicative that SARS-CoV-2 can be classified as a hepatotropic virus [60]. Indeed, in vitro studies have demonstrated that hepatocytes express ACE-2 receptors and transmembrane serine protease, which are crucial for viral entry. Interestingly, the entry and replication of SARS-CoV-2 is without cytopathic changes. Studies indicate that the virus promotes hepatic glucose production in the hepatocytes through the gluconeogenic pathway [61]. This, therefore, can, in part, explain the hyperglycaemia observed in COVID-19. Mechanistically, molecular analysis suggests the virus promotes the activity of the key gluconeogenic enzyme, phosphoenolpyruvate kinase (PEPCK) [62]. PEPCK is a rate-limiting enzyme that is inhibited by insulin whilst stimulated by glucagon and other hormones, including glucocorticoids and catecholamines. Studies have reported that the activity of PEPCK was not associated with genetic transcription [61]. This, therefore, suggests that SARS-CoV-2 may modulate this enzyme through acetylation, thus promoting its activity. In a COVID-19 context, the virus aims to promote its propagation and host weakness by ensuring glucose availability in infected immune cells [63,64]. To efficiently utilise glucose to its benefit, SARS-CoV-2 has been reported to reprogram glucose metabolism, favouring the glycolytic pathway over oxidative phosphorylation [65]. The reprogramming involves forcing the infected cells, particularly monocytes, to oxidise glucose through glycolysis for immediate ATP generation to support cell proliferation and the synthesis of molecules of pro-inflammatory cytokines [66]. To ensure glycolytic oxidation dominates, oxidative phosphorylation, the virus has been reported to induce mitochondrial oxidative damage. Furthermore, studies have reported increases in lactate dehydrogenase over pyruvate dehydrogenase, further supporting the metabolic shift toward hyperglycolysis [67]. From a biochemical perspective, favouring hyperglycolytic pathways could ensure the sustainable production of lactate [62]. Lactate, amongst other metabolic substrates, including alanine and glutamates, are the main precursors for glucose in gluconeogenesis. Hyperglycolysis could, therefore, afford the availability of the substrates required for the hepatic gluconeogenic pathways, which is a major source of glucose. In a COVID-19 setting, the glycolytic pathway may also be induced by hypoxic conditions either due to limited ventilation or septic shock due to a cytokine storm. The increases in gluconeogenesis could, therefore, be part of the mechanisms associated with SARS-CoV-2-induced hyperglycaemia. Furthermore, the overproduction of lactate could also be associated with the induction of an insulin resistance state, which promotes the development of T2DM. Studies have reported that direct exposure to lactate to insulin-sensitive cells induces insulin resistance [68]. Contrary to our argument, clinical studies analysing the acid–base balance in COVID-19 reported a lack of plasma lactate accumulation [69]. The possible explanation behind this observation was ascribed to the utilisation of lactate in the gluconeogenic pathway [66].

## 8. SARS-CoV-2 and Inflammation

SARS-CoV-2 infection elicits an immune response due to the damage in the respiratory system. This response calls upon specialised immune cells like macrophages and monocytes to combat the infection. These cells not only combat the virus but also release signalling molecules called cytokines, which, in turn, prepare the body’s immune system by activating T and B-cells for a more tailored defence. During infection, the virus also triggers the activation of specific host Toll-like receptors (TLRs), leading to the release of a wide range of pro-inflammatory cytokines. These include interleukin IL7, IL-2, IL-6, interferon-gamma (IFN-γ), and TNF-α [41]. However, this response can become dysregulated and will result in a phenomenon known as a cytokine storm, which results in extensive inflammation in the lungs [70]. The cytokine storm caused by COVID-19 is not only localised to the lungs, but it causes widespread systemic inflammation, which damages different organs, including the insulin-producing organ, the pancreas [71].

**Figure 2:**
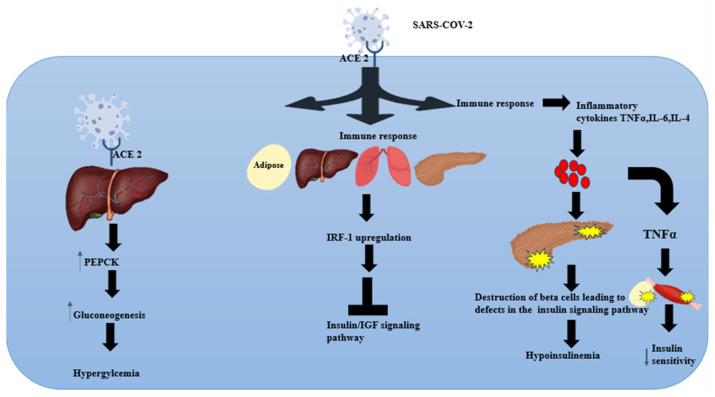
A depiction of the possible mechanisms of how the SARS-CoV-2 virus potentially results in new-onset diabetes. The possible mechanisms are pancreatic β-cell destruction, TNF-induced insulin resistance in the skeletal muscle and adipose tissue, as well as sustained hyperglycaemia due to hepatic gluconeogenesis [42,62,65,75,76,81].

A cytokine storm is an exaggerated and uncontrolled immune response that results in many cytokines, such as IL-6, IL-4, and TNF-α, being released in a short period, resulting in widespread inflammation and tissue damage [72]. According to Chen et al., COVID-19 patients who died had higher levels of IL-2 receptors, IL-10, TNF-α, IL-6, and IL-8 when compared to those who recovered [72]. In a study conducted by Queiroz M et al., the findings indicated that a long COVID-19 cytokine profile appeared to be characterised by high levels of IL-17 and IL-2 and low levels of IL-4 and IL-10 [73]. The take-home message is that elevated cytokine levels lead to multiple organ system failure as endothelial dysfunction, metabolic dysregulation, and vascular damage occur [74]. Studies conducted both in vitro and in vivo have demonstrated that the increased expression of TNF-α is linked to a reduction in insulin sensitivity in both skeletal muscle and adipose tissue [75,76,77]. This can be postulated to be a propagating agent in the development of new-onset diabetes. Furthermore, increased levels of TNF-α have also been shown to cause conditions like septic shock and the failure of multiple organs [70]. Steinberg et al. also reported that elevated TNF-α would cause insulin resistance via a decrease in insulin sensitivity in the skeletal muscles and adipose tissue [77]. Another study conducted on healthy human subjects by Ploomgaard et al. also confirmed that TNF-α induced skeletal muscle resistance [76]. The clinical significance of this study’s data was that even a small increase in the TNF-α concentration could negatively affect peripheral insulin action and also affect the ability of skeletal muscle cells to store glucose in response to insulin [76]. This phenomenon was observed because TNF-α decreases GLUT4 expression, which is essential for glucose transport in insulin-sensitive tissues [78]. Since 1997, it has been hypothesised that chronic low-grade inflammation is closely linked to the aetiology of diabetes [71]. Since that time, over a dozen studies have revealed that pro-inflammatory cytokines are responsible for the body’s acute-phase response and that IL-6, TNF-α, and interferons present in the bloodstream can serve as reliable indicators for predicting the onset of T2DM [55,71,79]. T2DM is linked to persistent low-level inflammation, and COVID-19 triggers an excessive inflammatory response; the interaction between these inflammatory conditions may amplify the harm caused by inflammatory agents [80]. As the immune response plays a substantial role in the evolution of COVID-19, assessing the inflammatory mediators at a cellular level may provide better insight into how the host’s response may be attributed to new-onset T1DM and T2DM. Given this background, Figure 2 shows the proposed potential mechanistic pathways that SARS-CoV-2 may potentially utilise to cause new-onset T1DM and T2DM.

## 9. COVID-19 and New-Onset Diabetes: Outcomes with New-Onset Hyperglycaemia with or without Diabetes in People Who Have Suffered from COVID-19

Due to the ongoing debate regarding the relationship between COVID-19 and diabetes, this table presents a brief overview of the current findings from various studies assessing the bidirectional relationship between the two. Further research should be conducted to fully elucidate the precise mechanisms underlying this relationship, ensuring a clearer understanding of the interaction between COVID-19 and diabetes.

## 10. Authors’ Perspectives and Recommendations

COVID-19, despite its devastating impact on human life, has, however, provided several lessons and has further accelerated our understanding of the aetiology and pathogenesis of diabetes mellitus. It has further solidified the impact of viral infections and their role in metabolic reprogramming in inducing glycaemic aberrations. Understanding the cellular and molecular mechanical insights pertaining to glucose metabolism is crucial as it consolidates the links between the diseases, therefore allowing us to contemplate preventative therapeutic interventions, not only for preventing diabetes but also for combating the virus itself. Understanding the metabolic reprogramming induced by SARS-CoV-2 enabled Santo et al. to suggest therapeutic strategies to overcome the virus. These strategies included GLUT-1 blockers and glycolysis inhibitors. In general, the metabolic derangement associated with SARS-CoV-2 aims to induce hyperglycaemia, which could also explain its heightened virulence in diabetic conditions. With a concrete link associated with SARS-CoV-2 infection and diabetes development, several strategic interventions should be conceptualised and implemented. At a minimum, healthcare authorities should recommend and intensify pre-diabetes and diabetes screening. In the last 4 years, it is plausible that significant pre-diabetic individuals converted to overt diabetes mainly due to SARS-CoV-2 infection. Furthermore, SARS-CoV-2 not only increases the risk of developing diabetes but can worsen its progression, thus accelerating the development of long-term complications such as diabetic nephropathy, neuropathy, and retinopathy, especially in unmanaged hyperglycaemia. The mechanisms we have highlighted in the preceding sections should also bring into light the question of whether anti-hyperglycaemics are still as effective in managing hyperglycaemia during or after post-SARS-CoV-2 infection. With risks of pancreatic damage during SARS-CoV-2 infection, a type 2 diabetes patient is at risk of losing the ability to produce and secrete insulin. In such a scenario, such patients would not benefit from sulphonyl urea-based therapy, and in this case, insulin therapy would suffice as a substitute. Similarly, having highlighted the risk of developing insulin resistance, type 1 diabetes patients may have a challenge in affording tight glycaemic control using insulin alone; in such a case, an insulin sensitiser may be required. Indeed, in the prior section, we have alluded to cases where higher insulin doses were required to achieve glycaemic control. From what we have highlighted above, clinicians and authorities should consider SARS-CoV-2 infection as a risk factor for the ineffectiveness of anti-hyperglycaemics. In conclusion, SARS-CoV-2 infection presents itself as a risk factor for developing diabetes mellitus, possibly through (1) the destruction of insulin-producing cells, (2) increased hepatic gluconeogenesis, and (3) insulin resistance through an elevated inflammatory state and lactate production.

## Figures and Tables

**Table 1:** Demonstrates the relationship between COVID-19 and diabetes mellitus development.

Study Reference	Study Design and Data	Key Findings, Associated Effects, and Remarks
Unsworth et al., 2020 [31]	Multi-centre regional study: data on new-onset type 1 diabetes for 30 children aged 23 months to 16.8 years that developed new-onset type 1 diabetes	The study supports the bidirectional relationship between COVID-19 and new-onset diabetes. This is the first report to outline a noticeable rise in the occurrence of new-onset T1D among children during the COVID-19 pandemic, with indications of SARS-CoV-2 infection or exposure identified in a portion of the tested individuals.
Müller et al., 2021 [32]	ACE2 pancreatic islet cells	SARS-CoV-2 infects and replicates in cultured human islets. This supports the bidirectional relationship between COVID-19 and new-onset diabetes.
Coppelli et al. [12]	Cohort study of 271 hospitalised COVID-19 patients with diabetes and no diabetes	COVID-19 patients who developed hyperglycaemia without having pre-existing diabetes had a higher mortality rate when compared to those with normoglycemia. Hyperglycaemia is indicative of a link between COVID-19 and diabetes.
Fadini et al. [33]	Retrospective study: 21 COVID-19 patients with new-onset diabetes	A higher rate of ICU admission and mortality among COVID-19 patients with new-onset diabetes than among those with pre-existing diabetes and normoglycemia was observed. New-onset diabetes had the worst prognosis out of the three. This paper supports the bidirectional relationship between COVID-19 and new-onset diabetes.
Singh and Khunti et al. [34]	Literature review	New-onset hyperglycaemia and new-onset diabetes have been increasingly recognised in the context of COVID-19.
Bode et al. [35]	Retrospective observational study	Individuals with new-onset hyperglycaemia have poorer outcomes than those with pre-existing diabetes. New-onset hyperglycaemia without diabetes had poorer outcomes than pre-existing diabetes.
